# EXAMINING THE IMPACT OF TRAUMA-INFORMED CARE ON NEUROPSYCHIATRIC SYMPTOMS IN THE LONG-TERM CARE SETTING

**DOI:** 10.1093/geroni/igae098.4178

**Published:** 2024-12-31

**Authors:** Sahebi Saiyed, Alexis Bender

**Affiliations:** Emory University, Atlanta, Georgia, United States; Emory University, Atlanta, Georgia, United States

## Abstract

Major life changes, such as transitioning into skilled nursing care, can trigger a traumatic stress response in older adults. In 2019, CMS released the requirement for trauma-informed care (TIC) in Skilled Nursing Facilities (SNF). This exploratory project aimed to assess the effectiveness of TIC on reducing neuropsychiatric symptoms, assessed using the NPI-NH, among patients with a history of trauma in one SNF. We conducted a retrospective chart review and trauma screening of 59 patients; primarily female (n=51, 86%), white (n=35, 59%) and ranged in age from 67-102 (Mean=82.97). 43 (73%) patients reported trauma and 27 (46%) patients were defined as “high risk” (i.e., history of trauma and either an NPI-NH score (≥ 20) or ≥2 psychotropic medications). 12 (44%) “high-risk” patients received a targeted care plan intervention (i.e., consistent staffing, music therapy, spiritual support, and specialized nursing care) based on the trauma reported and input from patients or their care partners. NPI-NH scores we reassessed one month after the intervention initiation. The NPI-NH score for the intervention group was 36.00 (SD=19.34) at baseline and 24.56 (SD=19.68) at follow-up. We conducted a paired samples t-test (pre- to post-intervention) with a more lenient p-value of 0.10 due to the exploratory nature of this intervention. We found there was a significant difference with a moderate effect between the mean NPI-NH scores from pre- to post-intervention, t(11) = 1.95, p=.08, Cohen’s d = 0.59, indicating promise for TIC interventions to improve the quality of life for patients admitted into SNF with a trauma history.

